# Epigenetic reprogramming in gastrointestinal cancer: biology and translational perspectives

**DOI:** 10.1002/mco2.670

**Published:** 2024-08-24

**Authors:** Yingjie Wang, Hongyu Liu, Mengsha Zhang, Jing Xu, Liuxian Zheng, Pengpeng Liu, Jingyao Chen, Hongyu Liu, Chong Chen

**Affiliations:** ^1^ State Key Laboratory of Biotherapy and Cancer Center West China Hospital Sichuan University Chengdu Sichuan China

**Keywords:** biomarkers, DNA methylation, epigenetic, gastrointestinal cancer, histone modifications

## Abstract

Gastrointestinal tumors, the second leading cause of human mortality, are characterized by their association with inflammation. Currently, progress in the early diagnosis and effective treatment of gastrointestinal tumors is limited. Recent whole‐genome analyses have underscored their profound heterogeneity and extensive genetic and epigenetic reprogramming. Epigenetic reprogramming pertains to dynamic and hereditable alterations in epigenetic patterns, devoid of concurrent modifications in the underlying DNA sequence. Common epigenetic modifications encompass DNA methylation, histone modifications, noncoding RNA, RNA modifications, and chromatin remodeling. These modifications possess the potential to invoke or suppress a multitude of genes associated with cancer, thereby governing the establishment of chromatin configurations characterized by diverse levels of accessibility. This intricate interplay assumes a pivotal and indispensable role in governing the commencement and advancement of gastrointestinal cancer. This article focuses on the impact of epigenetic reprogramming in the initiation and progression of gastric cancer, esophageal cancer, and colorectal cancer, as well as other uncommon gastrointestinal tumors. We elucidate the epigenetic landscape of gastrointestinal tumors, encompassing DNA methylation, histone modifications, chromatin remodeling, and their interrelationships. Besides, this review summarizes the potential diagnostic, therapeutic, and prognostic targets in epigenetic reprogramming, with the aim of assisting clinical treatment strategies.

## Graphical abstract

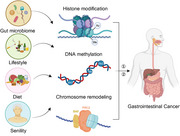

## 1 INTRODUCTION

The development of cancer arises from the dysregulation of fundamental biological processes in the organism, such as cell proliferation, cell death, cell invasion, and cellular metabolism.^1^, ^2^ Throughout the extensive timeline of cancer research, it has been widely believed that genetic mutations are responsible for disrupting these biological processes, thereby establishing the prominent role of cancer genetics. Nevertheless, recent years have borne witness to the acknowledgment of epigenetic reprogramming as a pivotal force in the instigation and advancement of cancer, posited as an alternative oncogenic mechanism.^3^ Epigenetic reprogramming refers to heritable and reversible changes in gene expression and chromosomal stability that occur without altering the underlying DNA sequence. Epigenetic dysregulation exerts its influence upon 3D chromatin structure, mediated through alterations in DNA methylation, anomalous histone modifications, posttranscriptional regulation by noncoding RNAs, and chromatin remodeling, thus orchestrating the modulation of pivotal genes intertwined with physiological or pathological processes.^4^, ^5^, ^6^ These epigenetic alterations do not act in isolation but rather cooperate through coordination or antagonism, collectively shaping the occurrence of tumors.

The whole genome and epigenome unveil the widespread occurrence of epigenetic reprogramming in cancer cells, revealing the breadth and depth of its alterations.^6^, ^7^ It is postulated that epigenetic alterations manifest at an early juncture in cancer, conceivably antecedent to genetic mutations. Evidently, the synergistic interplay between genetic perturbations and epigenetic reprogramming collectively sculpts the initiation and progression of cancer. Illustratively, recent revelations concerning oncohistones elucidate instances where histone mutations emanate from genetic imbalances, concurrently exerting influence on histone modifications and effecting a functional reshaping of chromatin structures.^8^, ^9^ Various environmental factors are deemed triggering agents, encompassing factors such as smoking, alcohol consumption, diet, age, and infections.^10^, ^11^, ^12^ Noteworthy is the dynamic nature of epigenetic reprogramming—a process where cells continually adapt DNA, histone, and noncoding RNA modifications to meet developmental requirements or respond to external stimuli. Moreover, the anomalous regulation of gene enhancers and super‐enhancers serves to further illuminate and deepen our comprehension of the role played by epigenetics in the context of cancer.^13^, ^14^

Gastrointestinal cancers are a huge threat to humanity, accounting for more than a quarter of all cancer incidence and more than a third of cancer‐related mortality.^15^ These malignancies, including esophageal, gastric, colorectal, liver, and pancreatic cancers, are marked by rapid progression, pronounced heterogeneity, and limited treatment efficacy.^10^ Owing to these characteristics, the overall 5‐year survival rate for patients with advanced gastrointestinal cancer remains below 15%, even with the integration of chemoradiotherapy and contemporary surgical techniques.^16^ Gastrointestinal cancers as a whole are on the rise, and experts predict that by 2040, new cases and deaths will reach 58 and 73% of all cancer patients. Epidemiological analyses reveal a regional distribution pattern, with the highest incidence and mortality rates observed in East Asia, particularly for gastric, liver, and esophageal cancers (ECs).^17^ This trend is likely attributable to population growth, aging, and lifestyle factors prevalent in the region.

**FIGURE 1:**
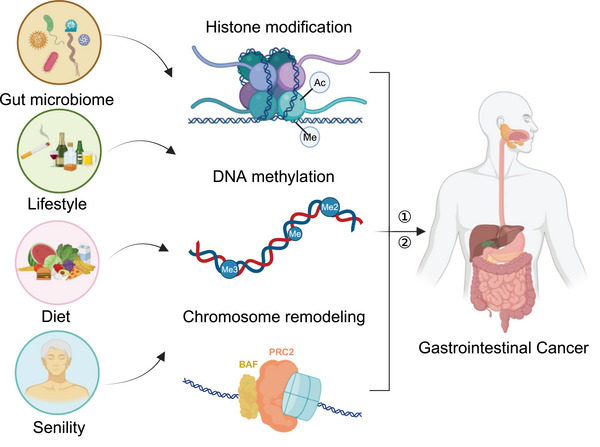
The overview of epigenomics in gastrointestinal cancer. Infection, smoking, alcohol, diet, senility and other factors affect the reprogramming of the epigenetic genome and then promote the occurrence and development of gastrointestinal cancer. ① indicates “function despondently” and ② indicates “interaction.”

Over the preceding decade, an abundance of scholarly investigations attests to the pivotal roles played by DNA methylation and histone modification in the orchestration of oncogene and tumor suppressor gene regulation, steering critical junctures in initiation and progression of gastrointestinal cancers (Figure 1). For example, this insight presents opportunities for targeted therapies with specific inhibitors. Aberrant DNA methylation of CpG islands (CGIs) in promoters, catalyzed by DNA methyltransferases (DNMTs), can trigger the development of gastrointestinal cancers. Furthermore, histone methylation can impact the degree of chromatin openness.^18^ Noteworthy are syndromes associated with gastrointestinal cancers, exemplified by Lynch syndrome, emanating from lineage‐specific epigenetic dysregulation.^19^, ^20^ This review elucidates the role of epigenetic reprogramming in the genesis and progression of gastrointestinal cancers, with a focus on esophageal, gastric, and colorectal cancers (CRCs). Clinical implications, including diagnosis, treatment, and prognosis, are explored, thereby providing the theoretical underpinnings for the formulation of targeted pharmaceuticals and clinical practice directives.

## 2 BASICS OF EPIGENETICS

### 2.1 DNA methylation

In the 1970s, scholars unveiled a novel epigenetic indicator: the methylation of cytosine residues within the CpG dinucleotide region, facilitated by DNMT, utilizing S‐adenosyl methionine (SAM) as the methyl donor.^21^, ^22^ Sustained involvement of DNMT1, DNMT3A, and DNMT3B proves imperative for methylation^23^ and is indispensable for embryonic or neonatal development.^24^, ^25^ These investigations underscore the pivotal role of 5‐methylcytosine (5mC) in mammalian differentiation. In mammals, 60−90% of CpG sites undergo methylation, while the residual unmethylated CpG clusters configure CGIs situated at the core sequences of promoters and transcriptional initiation sites.

Various methodologies have been devised to explore methylation sites throughout the genome, including the utilization of 5mC capture via methylation‐sensitive restriction enzymes or methylated DNA‐binding proteins, followed by sequencing.^26^ DNA methylation primarily takes place in repetitive genomic domains and serves as a crucial mechanism for maintaining overall genomic stability.^27^ Interestingly, CGIs, found within the promoter region, are rarely subject to methylation. Distinct genomic methylation locations exert diverse influences on gene expression. Methylation events occurring in the gene itself often contribute to enhanced gene expression, while methylation occurring in the promoter region of the CGIs may lead to transcriptional suppression of the genes involved.^28^ Moreover, it has been postulated that genomic methylation might impact chromosome remodeling; for instance, methylation of repetitive regions such as the centromere plays a pivotal role in chromosome stability.^29^

### 2.2 Histone modification

The nucleosome, composed of chromosomal DNA and a histone octamer, serves as the fundamental repetitive entity of chromatin. In the nucleus of eukaryotic cells, numerous nucleosomes are bound into compact higher‐order chromatin structures by linker DNA.^30^ Chromatin‐modifying enzymes selectively recruit specific effector proteins to the N‐terminal tails of histones to facilitate posttranslational modifications (PTMs), thereby influencing chromatin architecture.^31^ PTMs, encompassing methylation, acetylation, ubiquitination, phosphorylation, sumoylation, and proline isomerization, among others, play a pivotal role in gene expression regulation and participate in various cellular biological processes. They can result in genomic instability, impaired DNA damage repair, and gene transcriptional inactivation, thereby inducing the occurrence and development of certain cancers, such as breast cancer, CRC, and gastric cancer (GC).^32^ Notably, recent advances in cancer epigenetics have demonstrated a massive reprogramming of histone modification that induces silencing of essential tumor suppressor genes and activation of proto‐oncogenes.^33^

**FIGURE 2:**
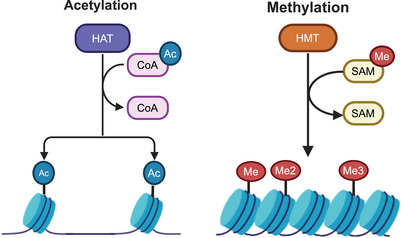
The most classical patterns of histone modification: histone acetylation and histone methylation.

Histone acetylation (Figure 2) assumes a pivotal role in governing the architecture and functionality of chromatin. Typically, active genes are associated with hyperacetylated histones, whereas deacetylated histones are found in silent regions of heterochromatin.^34^ Histone acetylation regulates DNA function through two mechanisms. First, the acetylation of lysine residues counters the positive charge of histones, attenuating the interaction between histones and DNA, thereby engendering alterations in chromatin configuration. Second, lysine modification serves as an anchorage point for the recruitment of transcription factors and chromatin modification proteins. Remarkably, histone acetylation levels are dynamically modulated by histone acetyltransferases (HATs) and histone deacetylases (HDACs). HATs neutralize the positive charge of chromosomal components, thus opening chromatin and promoting gene expression, while HDACs condense chromatin and silence gene expression. HATs can be classified into two categories on the basis of sequence homology, shared structural attributes, and functional roles: the GCN5‐related N‐acetyltransferases family, exhibiting the ability to acetylate lysine residues of H2B, H3, and H4, and the MYST family, distinguished by its highly conserved MYST domain. HDACs can be categorized into four classes, predicated on the homology with yeast HDACs: class I (HDAC1, 2, 3, 8), class II (HDAC5, 6, 7, 9, 10), class III (SIRT1‐7 and sirtuins), and class IV (HDAC11).^35^ Abnormal expression of HDACs can disrupt the balance of cellular biological systems, leading to the dysregulation of multiple genes and the development of cancer.

Histone methylation (Figure 2) is a fundamental epigenetic mechanism that is tightly controlled by the coordinated activities of histone methyltransferases (HMTs) and histone demethylases (HDTs), which facilitate the remodeling of chromatin structure and thereby modulate DNA replication and gene expression.^36^ HMTs and HDTs selectively add and remove methyl groups from specific amino acid residues on histones, with lysine (K) and arginine (R) being the most commonly targeted residues, while histidine (H) is infrequently methylated.^37^ Lysine methylation can manifest as mono‐, di‐, or tri‐methylation (Kme1, Kme2, or Kme3), whereas arginine can undergo mono‐ (Rme1s), symmetric di‐ (Rme2s), or asymmetric di‐methylation (Rme2a). HMTs are recruited to specific sites through their SET domains, which recognize lysine substrates and cofactors during catalytic events. HDTs encompass demethylases containing amine oxidase‐like domains and Jumonji C domains.^38^ Unlike histone acetylation, histone methylation does not influence the charge of the modified residues. The impact of histone methylation on gene expression varies depending on the specific location and degree of modification. For instance, methylation of H3R2, H3R8, and H3R17 is associated with transcriptional activation, while H3K9me3 and H3K27me3 are recognized as transcriptional repression markers.^39^

### 2.3 Chromatin remodeling

Each human cell contains over 3.2 billion DNA base pairs, which are compressed into higher‐order chromatin structures. The orchestration of these structures proves indispensable for the precision of gene expression.^40^ In this finely tuned process, the paramount significance of ATP‐dependent chromatin remodeling harmonizes with the earlier‐discussed DNA and histone modifications. These collaborative factors meticulously sculpt the chromatin structure, orchestrating the modulation of gene expression, thereby intricately influencing cellular fate and contributing significantly to the initiation and progression of cancer. Chromatin remodeling complexes can modify chromatin accessibility, resulting in either chromatin “opening up” or “compression.” Chromatin remodeling complexes rely on the hydrolysis of ATP to generate energy for their remodeling functions. The core subunit of these complexes is the ATPase catalytic subunit, based on which chromatin remodeling complexes are categorized into four classes: switch/sucrose nonfermentable (SWI/SNF), ISWI, CHD, and INO80/SWR1.^41^

Although the ATPase catalytic subunits in different classes share common functional domains, they concurrently possess unique amino acid sequences and domains. For instance, the N‐terminus of CHD‐type ATPases includes a chromodomain structure that can recognize and bind to lysine‐methylated histone H3.^42^ While CHD1 and CHD2 chromatin remodeling factors can autonomously execute remodeling functions, the prevailing modus operandi for the majority of remodeling complexes in vivo involves functioning as multisubunit entities. Historical investigations have unveiled a pivotal facet of chromatin remodeling complexes—acting as DNA translocases. This role facilitates nuanced alterations in the relative positions of DNA along the histone surface.^43^ In addition to this, they also function as executors of histone variant exchange into (or out of) nucleosomes, with a typical example being Swr1 in yeast, catalyzing the replacement between the H2AZ–H2B heterodimer and the classical H2A–H2B heterodimer within the nucleosome.^44^

## 3 COMMON EPIGENETIC CHANGES IN GI CANCERS

Epigenetic reprogramming is prevalent in both malignant and premalignant tumors of the gastrointestinal tract and is intricately linked to angiogenesis, tumor growth, and prognosis.^45^ Epigenetic reprogramming perturbs the equilibrium between oncogene and tumor suppressor gene transcription by modulating DNA methylation, histone modification, and chromatin remodeling, thereby driving tumor progression, invasion, and resistance to novel therapeutic strategies.^46^ Comprehensive DNA hypomethylation and abnormal regional DNA hypermethylation are characteristic of all gastrointestinal cancers, with DNA hypermethylation promoting carcinogenesis by silencing tumor suppressor genes.^47^, ^48^ Histone modifications, such as *EZH2*‐mediated methylation of H3K27, play a critical role in the malignant transformation of precancerous lesions into gastrointestinal cancers.^49^ Notably, these various epigenetic modifications function synergistically, forming a complex network that governs gene regulation.

### 3.1 DNA methylation

**FIGURE 3:**
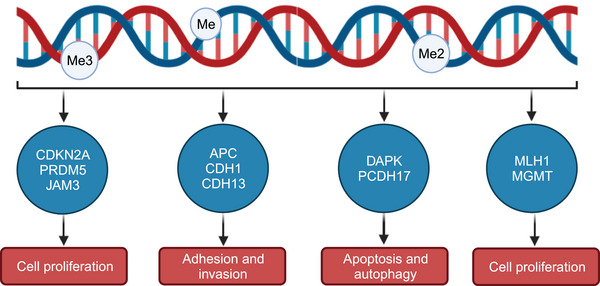
Examples of genes affected by hypermethylation or hypomethylation in gastrointestinal cancers, along with the associated signaling pathways involved.

Extensive alterations in DNA methylation patterns, characterized by genome‐wide hypomethylation and hypermethylation, have been observed in various types of cancer.^27^, ^50^ In gastrointestinal tumors, CGI methylation phenotype (CIMP) has been identified, characterized by abnormal, pervasive, and genome‐wide DNA hypermethylation of CGIs. For instance, CIMP has been described in GC,^51^, ^52^ liver cancer,^53^, ^54^ CRC,^55^ duodenal adenocarcinoma,^56^ and others. CIMP displays distinctive tissue‐specific methylation patterns across varied tumor types.^57^ CRC has mainly two CIMP subtypes, one driven by BRF4 mutation (53%) and the other by *KRAS* mutation (92%), both independent of age, gender, or staging.^55^ Analogously, the prevalence of CIMP has been identified in a substantial proportion of GC patients, ranging from 24 to 47%.^58^ Studies suggest the existence of two distinct CIMP groups in GC—“gastric CIMP” associated with microsatellite instability (MSI) and “Epstein–Barr virus (EBV) CIMP” associated with EBV positivity.^59^, ^60^ However, at the pathway level, various cancers with CIMP share some common features, such as the enrichment of polycomb repressive complex 2 (PRC2) targeting.^61^, ^62^ In addition, critical tumor suppressor genes involved in gastrointestinal cancer are epigenetically silenced via DNA methylation in the promoter region, leading to dysregulation of various cellular pathways including cell proliferation, adhesion, invasion, apoptosis, autophagy, DNA damage repair, and others (Figure 3).^50^, ^63^, ^64^ This dysregulation serves as a driving force behind the initiation, progression, migration, and invasion of tumors. Subsequent sections will delve into the genes that are commonly abnormally methylated in different cellular pathways in gastrointestinal tumors.

#### 3.1.1 Cell proliferation genes

Cyclin‐dependent kinase inhibitor 2A (*CDKN2A*), a member of the cyclin‐dependent kinase inhibitors family, exerts its function by inducing cell cycle arrest in the G1 phase, while simultaneously exhibiting hypermethylation characteristics in gastric adenocarcinoma.^65^ Throughout the process of gastric carcinogenesis, the frequency of *CDKN2A* methylation increases significantly, ranging from noncancerous dysplasia (4%) or adenoma (18%) to cancer‐associated dysplasia or adenoma (29%), ultimately leading to the development of gastric adenocarcinoma (44%). These observations suggest that *CDKN2A* plays a critical role in the malignant transformation of gastric precursor lesions. Similarly, high methylation of *CDKN2A* is significantly associated with somatic mutations in esophageal squamous cell carcinoma (ESCC).^66^

PE/SET Domain 5 (*PRDM5*), a member of the PRDM (PR‐domain‐containing) zinc finger gene family, possesses the capability to induce G2/M phase arrest and apoptosis in tumor cells, resulting in the inhibition of tumorigenesis.^67^ However, it has been duly reported that the precise methylation occurrence within its promoter region precipitates the unfortunate inactivation of *PRDM5* in a substantial proportion of GC cases.^68^ *PRDM5* also plays a role in the epigenetic regulation of oncogenes by recruiting histone‐modifying enzymes such as HMT G9a and HDAC 1.^69^ Members of the Junctional Adhesion Molecule (*JAM*) family (*JAM1, 2, 3*) all possess a conserved PDZ domain.^70^ Methylation within the promoter region of *JAM3* has been observed by certain scholars in 26.5% (13 out of 49) of precancerous lesions associated with esophageal carcinoma and in 51.1% (388 out of 760) of primary esophageal carcinomas, thereby modulating the expression of *JAM3*. In esophageal carcinoma, *JAM3* induces G1/S arrest, inhibits the Wnt pathway to suppress cell proliferation, and promotes apoptosis. *JAM3* also functions as a tumor suppressor in CRC. The methylation level of *JAM3* increase in four CRC cell lineages. Notably, the methylation status of *JAM3* exhibits no correlation with patient gender, age, alcohol consumption, or smoking habits.^71^

#### 3.1.2 Adhesion and invasion genes

Migration and invasion through the hematogenous pathway exemplify fundamental attributes of malignancy. The genes *APC*, *CDH1*, and *CDH13* play a pivotal role in metastasis, all of which exhibit promoter hypermethylation in GC.^72^ Adenomatous colorectal polyposis (*APC*), a negative regulator of the WNT signaling pathway, rarely undergoes mutations but is frequently inactivated (75%) through methylation in GCs of the intestinal type.^73^, ^74^ The hypermethylation of *APC* disrupts the degradation of β‐catenin, leading to its accumulation in the cytoplasm and subsequent activation of the WNT/β‐catenin signaling cascade, thereby fostering invasion of GC cells.^75^ Patients with a normal esophagus did not exhibit promoter methylation of *APC*. In contrast, Barrett's esophagus (BE) without dysplasia or with low‐grade dysplasia demonstrated significantly elevated promoter hypermethylation in mucosal biopsies, with rates of 31 and 50%, respectively (*p *< 0.01). Moreover, individuals afflicted with high‐grade dysplasia or adenocarcinoma displayed even greater frequencies of *APC* promoter hypermethylation, reaching 54 and 68%, respectively (*p *< 0.001). These findings suggest that the high methylation of *APC* is an early event in the progression of BE‐associated tumors.^76^ Furthermore, it is noteworthy that specific genes exhibit cancer‐type‐specific methylation patterns, with *APC* remaining unaltered in the context of human CRC.

Cadherin‐1 (*CDH1*) encodes the vital E‐cadherin protein, which assumes a pivotal role in orchestrating intercellular interactions and establishing cellular polarity.^77^ Recent investigations have unveiled the presence of distinct methylation patterns in the CGIs and 5′‐shore regions of the *CDH1* promoter in both normal gastric tissues and GC cells.^78^ In fact, *CDH1* inactivation transpires in approximately 50% of all GCs and in 83% of diffuse‐type GCs, primarily due to gene deletion or promoter hypermethylation.^79^ This inactivation event precipitates cancer cell detachment from the surrounding stroma, thereby facilitating migration and invasion of GC cells. Additionally, researchers, incorporating 1633 samples, identified a significant correlation between elevated *CDH1* methylation and the tumor status, lymph node involvement, and metastasis in esophageal adenocarcinoma (EAC).^80^

#### 3.1.3 Apoptosis and autophagy genes

Death‐associated protein kinase (*DAPK*) is an enzyme reliant on Ca^2+^/calmodulin and exhibiting serine/threonine kinase activity.^81^ Functioning as an apoptotic regulator, *DAPK* can instigate programmed cell death. The prevalence of *DAPK* methylation in GC stands at 42%, significantly linked to chemotherapy resistance in patients with metastatic or recurrent GC, consequently leading to an unfavorable prognosis. In CRC, *DAPK* also exhibits high methylation. Research indicates that, in comparison with normal mucosa, the detected levels and frequency of *DAPK* methylation are elevated in peri‐cancerous noncancerous mucosa. However, it is noteworthy that the elevated methylation levels of the *DAPK* gene hold statistical significance solely within the tumor tissue.^82^ Scholarly inquiries delineate a gradual ascent in the methylation gradations of *DAPK*, spanning from nontumorous epithelial regions in the environs of ESCC to intraepithelial neoplasia and advanced ESCC, demonstrating a notable correlation with *P53* mutations. This suggests that the methylation of *DAPK* contributes to the progression of dysplastic carcinogenic sequences in the ESCC carcinogenesis process.^83^ Nonetheless, the methylation of *DAPK* do not afford prognostic acumen concerning the responsiveness of individuals harboring locally advanced EC to neoadjuvant chemotherapeutic interventions.^84^

Procadherin 17 (*PCDH17*) exerts inhibitory effects on the proliferation of gastric carcinoma cells by inducing apoptosis and autophagy.^85^ Several investigations have reported frequent and specific methylation of the *PCDH17* promoter in GC, leading to its downregulation or silencing, thereby implicating *PCDH17* in gastric carcinogenesis. Noteworthy is the impact of *PCDH17* expression on the TNM staging of GC, thus rendering it a promising candidate as a putative tumor marker.^86^ Similarly, *PCDH17* undergoes silencing and methylation in almost all CRC cell lines and approximately 95% of primary tumors. However, the absence of *PCDH17* is only detected in 12% of colon cancer tissues. The protein expression of *PCDH17* is significantly correlated with the tumor staging and lymph node metastasis in CRC. The restoration of its expression can inhibit tumor growth both in vivo and in vitro by promoting apoptosis.^86^ In ESCC, *PCDH17* is also silenced through mechanisms involving either homozygous deletion or promoter methylation. Substantial methylation of the *PCDH17* promoter markedly associates with a compromised differentiation status in ESCC cells. The resurgence of its expression attenuates both cellular proliferation and migration.

#### 3.1.4 DNA damage repair genes

MutL homolog 1 (*MLH1*), a DNA mismatch repair (dMMR) gene, is frequently inactivated in GC due to promoter methylation. Elevated levels of *MLH1* promoter methylation result in impaired dMMR, which is associated with significant genomic instability. This instability is believed to contribute to the majority of cases of MSI in GC.^87^, ^88^ Conversely, when *MLH1* expression remains unchanged, tumors tend to progress through CIN pathway. *MLH1* methylation in somatic cells can lead to mismatch repair deficiencies in CRC.^89^ Analogously, investigators conducted a genetic scrutiny involving 157 instances of CRC, wherein *MLH1* promoter hypermethylation constituted 66%. Of significance is the observation that the remaining 18% of tumors manifested constitutional pathogenic variations (Lynch syndrome), while 11% displayed pathogenic variations in both alleles of somatic mismatch repair genes. This suggests that in the occurrence and development of CRC, both genetic and epigenetic factors play a coordinated role, influencing the direction of cancer development. In contrast to gastric and colorectal malignancies, elevated methylation of the *MLH1* promoter is infrequent in EAC.^90^, ^91^ Nevertheless, recent investigations have unearthed a substantial correlation between *MLH1* promoter methylation levels and MSI‐H status in tumors. The study scrutinized 19 nondysplastic BE specimens and 145 EAC samples, exposing pronounced methylation of the *MLH1* promoter in all MSI‐H cases, with only one tumor displaying *MLH1* hypermethylation in MSS. It is noteworthy that the level of *MLH1* methylation in BE samples was significantly lower than in EAC samples, suggesting that *MLH1* may influence the premalignant nature of BE‐type lesions.^92^

*MGMT*, also known as O‐6‐methylguanine‐DNMT, plays a pivotal role in DNA repair by eliminating mutagenic alkyl groups from the O6 position of guanine.^93^ Prior investigations have documented a prevalence of MGMT hypermethylation in nearly 30% of gastric malignancies, precipitating the loss of *MGMT* protein and consequent genomic instability.^94^ Previous research has reported a significant risk of *MGMT* gene DNA hypermethylation in tumor tissues of individuals homozygous for MTHFR C677T (TT), with an OR (95% CI) of 3.15.^95^ Notably, in the majority of GC samples, hypermethylation of *MLH1* and *MGMT* promoters does not occur simultaneously.^96^ Similarly, a substantial prevalence of *MGMT* promoter methylation, accounting for 57%, is observed in EAC patients.^97^ Research has identified a significant correlation between high methylation of the *MGMT* promoter and tumor location in EAC patients (*p *= 0.0070).^98^ It is of significance to highlight that *MGMT*, by virtue of its promoter hypermethylation, emerges as a discernible marker for sporadic CRC.^99^

### 3.2 Histone modification

Histone modification is another epigenetic modification that plays an important role in the development, progression, metastasis, and drug resistance of gastrointestinal tumors. Histone methylation is a reversible PTM of histones, which regulates gene expression, chromatin structure reconstruction, and DNA damage repair.^100^ Lysine‐specific demethylase 1 (LSD1) is the first human HDT identified, and lysine‐specific demethylase 2 (LSD2) is its homologue.^101^ LSD1's control of histone demethylation is a key component of gastrointestinal tumorigenesis mechanism, such as its action on the well‐known tumor suppressor protein P53.^102^ Recent investigations have spotlighted the dysregulated expression of LSD2 as a frequent accomplice in perturbing histone dynamics, consequently engendering aberrant gene expression profiles implicated in various malignancies such as gastric, breast, and pancreatic cancers.^103^ Notably, histone methylation is one of the important components of the gastrointestinal epigenetic network, rather than acting alone. Multiple tumor‐associated lncRNAs have been found to regulate cancer progression through interactions with *EZH2* and *LSD1*.^104^

Metastatic competence in tumor cells hinges upon the acquisition of pivotal traits encompassing proliferation, migration, and invasion, wherein histone acetylation assumes a pivotal regulatory role.^105^ Noteworthy examples include the observed enhancement of CRC cell proliferation, migration, and invasiveness consequent to HDAC1 overexpression, countered by the abrogation of these malignant attributes upon HDAC1 silencing.^106^ Numerous sequencing results also found that tumor variation in ESCC was positively correlated with levels of histone acetylase H3K18ac.^107^ Mechanistically, H3 histone deacetylation attenuates the activity of cyclin‐dependent kinase inhibitor P21, while hypermethylation of the *ZNF312b* oncogene potentiates the progression of gastric tumors.^108^ Collectively, these studies have shown that different levels of histone acetylation are strongly associated with the development of gastrointestinal cancers.

It is noteworthy that the modification processes of all histones require metabolites. These include acetyl‐CoA, nicotinamide adenine dinucleotide, SAM, α‐KG, flavin adenine dinucleotide, ATP, and succinic acid, serving as substrates or cofactors. Metabolic reprogramming, exerting influence on macromolecular biosynthesis and energy production, actively contributes to the orchestration of histone modifications, dynamically altering the expression patterns of genes.^40^ For instance, the elevation of succinate inhibits HDTs, increases trimethylation of H3L4, thereby triggering the expression of tumor‐specific genes.^109^ In the progression of pancreatic ductal adenocarcinoma (PDAC), the oxidative pentose phosphate pathway orchestrated by 6‐phosphogluconate dehydrogenase facilitates the reprogramming of histone H3K9, propelling the transcription of n‐cadherin and promoting distant metastasis mediated by n‐cadherin.^110^ Short‐chain fatty acids (SCFAs), the principal metabolites generated by the fermentation of insoluble dietary fibers by gut microbiota, can inhibit HDAC. SCFA‐guided regulation enhances the response of CRC patients to chemotherapy and immunotherapy.^111^ Moreover, metabolites can directly serve as histone‐modifying enzymes. Butyrate, generated through the fermentation of dietary fibers by the gut microbiota, acts as a HDAC inhibitor (HDACi), demonstrating a protective role in gastrointestinal cancers.^112^

Lactic acid is a metabolic byproduct of glycolysis and possesses various crucial physiological and pathological functions. In 2019, Zhang et al.^113^ discovered a novel epigenetic modification called lysine lactylation, utilizing histone lysine lactylation to regulate gene expression in macrophages. Lactate dehydrogenase A (LDHA), a pivotal glycolytic enzyme, demonstrates upregulated expression across an array of gastrointestinal malignancies, encompassing gastric, esophageal, and pancreatic cancers. Liver metastasis is commonly detected in CRC. Recent research has revealed that *GPR37* can enhance glycolysis and H3K18la lactylation through the Hippo pathway, promoting liver metastasis in CRC.^114^ Lactate also serves as a primary epigenetic carbon source for histone acetylation. In pancreatic cancer, ^13^C3‐lactate carbon is used for acetylation of histone H4, a process that is dependent on nucleologically localized LDHA.^115^ These studies collectively indicate that lactate plays a major structural and regulatory role in the metabolism–epigenetic reprogramming axis, necessitating further exploration to unearth its clinical potential.

Apart from metabolism, viral infections also exert regulatory effects on chromatin modifications. Epstein–Barr virus (EBV), a human gamma herpes virus, is incapable of integrating into the host genome, yet it can engage in interactions with it. Multiple studies have substantiated that the interaction between EBV fragments and the host genome leads to the redistribution of histone marks. Notably, a particular investigation unveiled that EBV infection significantly elevates the levels of H3K27ac in the enhancer region while simultaneously reducing the level of H3K27me3 surrounding the transcription start site (TSS) in gastric epithelial cells.^116^ Aberrant histone marks are predictors of poor prognosis in nasopharyngeal and GCs. In addition, the loss and rearrangement of heterochromatic histone marks, leading to the silencing of the C promoter (Cp), is one of the EBV‐immortalized B lymphoblastoid cell line's hallmark. EBV‐mediated transformation also triggers a genome‐wide decrease and redistribution of heterochromatic histone marks. Collectively, these findings imply that EBV infection induces the reprogramming of the cellular epigenome through aberrant histone modifications, ultimately exerting an impact on gene expression.

In recent years, an unexpected discovery based on The Cancer Genome Atlas is that direct targets of chromatin‐modifying enzymes (i.e., histones) undergo mutations in cancer, referred to as oncohistones.^117^ It is increasingly apparent that oncohistones mutations exert a direct influence on chromatin modification, inducing alterations in the epigenomic land, instigating irregularities in gene transcription, and influencing the repair of DNA damage, thereby fostering carcinogenesis. The initially identified histone mutations associated with cancer occur in the tail domain or vicinity of histone H3, typically involving PTMs K27 and K36 in the H3 variant H3.3. The oncohistone H3 tail can inhibit the function of homologous histone writers binding to oncohistones, leading to disturbances in the epigenetic and transcriptional states. Researchers, including Bagert et al.^118^ and others,^117^ have also indicated that many histone mutations found in cancer actually occur in the globular (i.e., core) domains, which are crucial for the integrity and stability of nucleosomes or DNA wrapping. Currently, the discovery of oncohistones is primarily focused on gliomas,^119^ sarcomas, and lymphomas.^120^ Recently, a novel histone mutation, H2BG53‐to‐D,^121^ was identified in PDAC. H2BG53D mutation weakens the interaction between nucleosome DNA and the histone octamer, enhances the expression of cancer‐related genes, and consequently intensifies the carcinogenic properties of PDAC. Despite the limited reports on oncohistones in gastrointestinal tumors, the insights gained from previous research suggest a potential significant role for oncohistones in the epigenetic reprogramming of gastrointestinal tumors, making them a prospective avenue for therapeutic interventions in affected patients.

### 3.3 Chromatin remodeling

A large number of studies have shown that chromosome remodeling can affect the occurrence and development of gastrointestinal cancers. Notably, AT‐rich interaction domain 1A (*ARID1A*) emerges as a pivotal constituent of the SWI/SNF chromatin remodeling complex, exerting regulatory dominion over a spectrum of neoplastic hallmarks encompassing invasion, metastasis, proliferation, apoptosis, and epithelial–mesenchymal transition (EMT). *ARID1A* is widely mutated in GC, EAC, liver cancer, CRC, bile duct epithelial cancer, and other gastrointestinal tumors.^122^ Research indicates that frequent mutations in chromatin remodeling genes *ARID1A*, *MLL3*, and *MLL* occur in 47% of GCs.^123^ The DNA repair protein MSH2 can recruit to gene loci through the SWI/SNF chromatin remodeler SMARCA4/BRG1, promoting enhancer–receptor interactions and subsequently regulating the cell adhesion pathway. Loss of *MSH2* in advanced GC is associated with reduced expression of the cell adhesion pathway, EMT, and enhanced tumorigenesis in vitro and in vivo.^124^ Similarly, SMARCA4 can be enlisted through *PRMT1*‐mediated H4R3me2a, amplifying EGFR signaling and propelling the advancement of CRC.^125^

Chromatin remodeling complexes can also increase cancer resistance to drugs. Research has elucidated that the excessive expression of *CHD4* exacerbates resistance to chemotherapy and augments cellular proliferation in GC patients.^126^ In ESCC, the amplification of *SOX2* not only reflects the selective maintenance of *SOX2* expression in tumor cells but also promotes significant evolution of chromatin remodeling and the *SOX2* cis‐regulatory element.^127^ These studies suggest that chromatin remodeling complexes play a crucial role in the development, metastasis, and drug resistance of gastrointestinal tumors, indicating their potential as therapeutic targets for cancer. Moreover, it is noteworthy that chromosome remodeling is not unilaterally deterministic; a plethora of investigations have elucidated the contributory roles of DNA methylation and histone modification in sculpting the chromosomal architecture.

## 4 EPIGENETIC CHANGES SPECIFIC TO DIFFERENT GASTROINTESTINAL CANCERS

Epigenetic reprogramming is pivotal in gastrointestinal tumor development, manifesting through genome‐wide DNA methylation aberrations like CIMP formation and abnormal histone modifications mediated by diverse factors, including metabolism. Notably, distinct gastrointestinal tumors exhibit unique epigenetic reprogramming profiles, elaborated upon subsequently.

### 4.1 Gastric cancer

GC ranks as the third most prevalent malignancy globally and the fifth leading cause of cancer‐related mortality, with patient survival contingent upon disease staging at diagnosis.^128^ Morbidity and mortality are regionally related, with higher incidence in China. Key risk factors encompass dietary patterns, tobacco use, and infection with Helicobacter pylori.^129^ Its high heterogeneity and difficult diagnosis lead to complicated treatment and prognosis of patients, which is one of the most challenging problems in medical oncology. Epigenetic reprogramming emerges as both an early oncogenic event and a late developmental phenomenon in gastric carcinogenesis,^130^ spanning the spectrum from gastritis and ulcers to metaplasia, dysplasia, and tumorigenesis. The absence of *CDH1* stands out as a significant hallmark of GC, with *CDH1* promoter methylation detected in 50% of hereditary diffuse gastric carcinomas, often collaborating with genetic mutations to silence the gene.^131^ Alterations in the activity of HATs and HDACs have been observed in GC. For instance, GC exhibits elevated expression of *HDAC1* and *HDAC2* genes. In normal cells, H4ac enhances the activity of tumor suppressor gene promoters, whereas tumor cells demonstrate a loss of acetylation. Furthermore, GC patients display reduced expression of the HAT TI60, which exhibits a close association with lymph node metastasis. Histone acetylation also assumes a critical role in TNM staging and the invasive of GC. It is worth emphasizing the extensive interconnection of histone modifications in GC, as suggested by Lavarone et al.,^132^ who propose that the global loss of H3K27 methylation facilitates abnormal accumulation of H3K27 acetylation through the facilitated diffusion of acetyltransferases into chromatin.

Research into histone methylation in GC has predominantly focused on H3 and H4. *EZH2*, a well‐known catalytic subunit of PRC2, is involved in the repression of target genes through the methylation of H3K27.^39^ *EZH2* knockdown inhibits cell growth and proliferation by affecting *RUX3* and *ANXA6*, while also promoting GC invasion and migration by altering the expression of *CDH1*.^133^, ^134^ Furthermore, *EZH2* downregulates the expression of *CXXC4*, thereby stimulating the activation of the Wnt signaling pathway in GC cells. *EZH2* is also implicated in the regulation of bivalent histone marks that govern the expression of multiple genes. A recent study has revealed the absence of a bivalent chromatin state in gastric adenocarcinoma.^135^ The histone lysine methyltransferase SET also impacts primary gastric tumor size, lymph node metastasis, TNM stage, and orchestrates various pivotal cancer‐associated genes and pathways, establishing it as a significant adverse prognostic factor for GC patients. Moreover, the SET and MYND domain containing protein 3 (SMYD3) plays a critical role in GC aggressiveness and holds promise as a prognostic target.^136^

### 4.2 Esophageal cancer

EC ranks eighth in global cancer incidence and sixth in cancer‐related mortality,^137^ constituting a significant public health concern. This malignancy manifests predominantly as ESCC and EAC, with ESCC representing approximately 90% of cases worldwide.^138^ Even if the operation or chemoradiotherapy of EC is relatively mature, the prognosis is still poor, and the 5‐year survival rate is about 20%. Gastroesophageal reflux disease, dietary patterns, and obesity are recognized as primary risk factors for esophageal malignancies.^25^ Furthermore, epigenetic alterations, including DNA methylation and histone modifications, intricately shape the landscape of molecular changes in EC. Many HDACs have been proven to be associated with the occurrence and development of EC, such as the abnormal expression of HDAC1, HDAC2, and HDAC3.^139^, ^140^, ^141^ The derangement of HDACs in EC precipitates a systemic perturbation in histone acetylation, resulting in the quelling of the expression of tumor suppressor genes. Emerging findings elucidate the overexpression of HDAC4 in advanced stages of ESCC, notably amplifying *CDK2/4* and CDK‐dependent Rb phosphorylation, fostering the proliferation of cancer cells, and attenuating patient survival rates.^142^ Aspirin promotes the death of cancer stem cells in ESCC by altering the level of histone acetylation and may serve as a potential adjuvant or preventive chemotherapy for ESCC.^143^

In addition to the conventional acetylation, a mounting body of evidence suggests a close association between the functions of HMTs EZH2, SMYD2, EHMT1,^144^, ^145^, ^146^ and HDTs LSD1, PHF8, KDM4C,^147^, ^148^, ^149^ and the occurrence of EC. Notably, patients with ESCC manifest significantly heightened expression of EZH2, correlating with tumor dimensions, distant metastasis, and abbreviated disease‐free survival. The heightened expression of LSD1 is intricately linked to lymph node metastasis and an unfavorable prognosis in EC, thereby identifying it as an innovative therapeutic target for EC.^149^ Additionally, research reports an increase in the expression of H3K27me2 in ESCC patients, associated with a higher tumor recurrence rate.^150^ It is of significance to highlight that lysine succinylation experiences substantial downregulation in ESCC cells. Scientific inquiry establishes that succinylation exerts a negative regulatory influence on histone methylation, thereby fostering ESCC metastasis.^151^

### 4.3 Colorectal cancer

CRC ranks as the third most prevalent malignancy globally, constituting over 10% of newly diagnosed cases annually. With a worldwide mortality rate of around 45%, there is an urgent need to develop effective treatments for CRC patients. Over the preceding two decades, investigators have unraveled the intricate association between distinct CRC‐specific patterns of gene expression and aberrant genetic activity. In 2015, researchers described the most clinically relevant and widely accepted subtypes of CRC.^152^, ^153^ Among these, CMS1 exhibited pronounced methylation, CMS3 displayed moderate methylation, while the remaining two categories demonstrated the lowest levels of methylation. Notably, in addition to the overall changes across the genome, there were also differences in methylation levels at local sites. In comparison with healthy colon epithelium, CRC exhibits upregulated expression of HDAC1 and HDAC2, which is associated with overall patient survival^154^; upregulated expression of HDAC3 is correlated with poor CRC differentiation.^155^ Investigative findings posit that HDAC2‐mediated H3K27 deacetylation represses the transcription of *ALKBH5*, consequently fostering the progression of CRC.^156^ Acetylation at K168/175 sites of KAT8 diminishes its binding activity, concurrently inhibiting the recruitment of RNA pol II to the promoter regions of ATGL and HSL. This downregulates lipid breakdown, impacting the invasive and migratory capabilities of CRC cells.^157^

The role of histone methylation in CRC has been subject to considerable research. MCT1, identified as a pivotal protein within the monocarboxylate transporter, manifests heightened K473 trimethylation in CRC, instigating neoplastic glycolysis and orchestrating M2 polarization in tumor‐associated macrophages. Remarkably, clinical investigations have elucidated a positive correlation between the augmentation of MCT1 K473 trimethylation and the progression of tumors, along with the overall survival rates in afflicted individuals.^158^ Lysine methyltransferase Suv4‐20h2, a critical regulator of epithelial plasticity, primarily governs the trimethylation of H4K20. Research indicates that the loss of Suv4‐20h2‐mediated H4K20me3 alters chromatin accessibility, compacts chromatin, and fosters the onset of CRC.^159^ These inquiries accentuate histone methylation as a propitious therapeutic target for CRC. Furthermore, H3K20me3 and H3K27me3 function as discerning biomarkers capable of delineating 49.2% of CRC cases.^160^

### 4.4 Liver cancer

In recent years, there has been a gradual increase in the incidence of liver cancer, which varies by ethnicity.^161^ Liver cancer mainly occurs in the context of liver cirrhosis, and due to smoking, excessive alcohol consumption, hepatitis virus infection, metabolic factors, and genetic factors,^162^ it has been found that DNA methylation detected in the blood of liver cancer patients is associated with mortality and may affect the development of liver cancer.^163^ In addition, methylation‐related enzymes can also undergo epigenetic reprogramming, which in turn promotes the occurrence and development of liver cancer.^164^ found that in liver cancer, the 5mC RNA methyltransferase NSUN7 is inactivated due to its DNA methylation, which further prevents proper mRNA methylation. In addition, *NSUN7* DNA methylation‐related silencing was associated with poor treatment response and clinical outcomes in HCC patients.

Numerous studies have shown that aberrant activity of histone modifications has been linked to liver cancer.^165^ Lactation of lysine has recently been identified in liver cancer, which occurs mainly on two tumor‐associated proteins, USP14 and ABCF1, which promote the development of liver cancer and lung metastasis.^166^ The researchers also found that the HMT protein G9a was significantly upregulated in HB cells. The pharmacological targeting of G9a significantly inhibited the growth of HB cells, organoids, and patient‐derived xenografts. These data suggest that G9a is a potential drug target for the treatment of HB and can also be used in combination with chemotherapy.^167^

### 4.5 Others

Most anal cancers are HPV‐associated squamous cell carcinoma (ASCC); adenocarcinoma accounts for less than 10% of anal cancers and originate from adenocytes orchronic fistulas. Melanoma and other cancers of the anal margin are very rare.^168^ In recent years, the incidence of anal cancer has been increasing, and preceded by high‐grade anal intraepithelial neoplasia (HGAIN; AIN2‐3). DNA methylation is associated with anal cancer. HGAIN samples show heterogeneous methylation patterns, and DNA methylation testing in biopsies can help diagnose HGAIN and anal cancer.^169^ Among them, the methylation levels of *ASCL1* and *ZNF582* increased with the severity of the disease (*p* < 0.00001).^170^, ^171^ In addition, the proliferation and differentiation of HPV‐positive anal cancer cells require the involvement of the histone deubiquitination enzyme USP46, which makes USP46 a target for the treatment of this type of cancer.^172^ Studies have also shown that loss of expression of the histone variant macroH2A2 is associated with the progression of anal tumors and can be used as a prognostic biomarker for high‐grade AIN and ASCC.^173^

Cholangiocarcinoma (CCA) is a group of highly heterogeneous epithelial malignancies that account for approximately 3% of gastrointestinal cancers. CCA can be either intrahepatic (iCCA) or extrahepatic. In recent years, the incidence and mortality of CCA, especially iCCA, have been increasing, and treatment options are quite limited. Almost all cases had no clinical symptoms before the age of 40 years, and their average age of diagnosis was 50 years.^174^, ^175^ Previous studies conducted a comprehensive analysis of the DNA methylation profile of CCA. A total of 98 hypermethylated genes and 93 hypomethylated genes have been identified, which are mainly related to the negative regulation of biological processes.^176^ Methylation levels of *RIP3*, *ITF2*, *RASSF1A*, *PTGS2*, *SOCS3*, *MINT2*, *TNFRSF10C*, and *DLEC1* were significantly increased, which was associated with *IDH1/2* mutations in iCCA. More than five out of eight of the methylated genes showed different clinicopathological features.^177^ In addition, HDAC1 expression is positively correlated with lymph node metastasis, vascular infiltration, advanced cancer, and poor prognosis.^178^ It has been reported that the expression of HDAC2 and HDAC8 in CCA tissues did not change abnormally, while the expression of HDAC3 in CCA tissues was significantly upregulated. Overexpression of HDAC3 can enhance the proliferation of CCA cells, inhibit *p53*‐induced apoptosis, and reduce the survival of patients.^179^

Gallbladder cancer (GBC) is the most common and aggressive tumor of the biliary tract.^180^ Early GBC is usually diagnosed by chance. GBC is generally diagnosed at an advanced stage due to its nonspecific symptoms and lack of sensitive screening tests. Patients with GBC have a lower removal rate (10–30%). GBC progresses from atypical hyperplasia to carcinoma in situ and finally to invasive carcinoma. Epigenetic inheritance affects the development and treatment of GBC. The hypomethylation and overexpression of *ABCB1/MDR1* and *ABCG2/BCRP* may play a potential role in tumorigenesis of GBC, especially in its early stages.^181^ In GBC, *PEBP1* is often downregulated and hypermethylated, and its methylation is significantly associated with lymph node metastasis and shorter survival.^182^ In addition, histone modifications affect the immune microenvironment of GBC. Li et al.^183^ found that niacinamide methyltransferase (NNMT) can promote the expression of IL6 and granulocytic‐macrophage transcription factor by reducing the trimethylation level of histone H3, thereby promoting the differentiation of macrophages into M2 tumor‐associated macrophages. This suggests that NNMT is a potential molecular target for GBC immunotherapy.^183^

Pancreatic cancer is a highly lethal malignancy of the digestive system with a 5‐year relative survival rate of 13%. It has the third highest death rate in the United States, for both women and men.^184^ The most common pathological type of pancreatic cancer is PDAC. The early diagnosis of pancreatic cancer is difficult due to its insidious onset and atypical early clinical manifestations. At diagnosis, only 10−20% of patients have resectable localized tumors, and about 50% have metastases.^185^, ^186^ However, it is important to note that even resectable PDAC has the potential to metastasize. PDAC exhibits extensive metabolic and epigenetic reprogramming, which regulate each other and play a role together in tumor development. The study of the epigenomic landscape has created an advantage for establishing PDAC subtypes.^187^, ^188^ The widely accepted classical basal‐like subtypes were established primarily by identifying epigenetic reprogramming. In addition, deletion of the HDAC *KDM6A* can induce squamous, metastatic pancreatic cancer by activating the super amplifiers of oncogenes, such as *RUNX3* and *MYC*.^189^

## 5 GLOBAL LANDSCAPE OF EPIGENETICS IN GASTROINTESTINAL CANCERS

**FIGURE 4:**
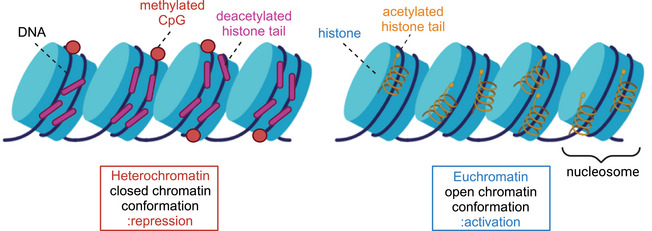
DNA methylation and histone modifications interact to shape euchromatin, heterochromatin.

Gastrointestinal cancer is characterized by a reprogrammed epigenome featuring genome‐wide hypomethylation, gene‐specific hypermethylation, and aberrant histone modifications.^18^ Epigenetic modifications are not isolated processes but instead exhibit significant interplay, with evidence demonstrating that changes in DNA methylation and histone modifications in cancer occur concurrently and depend on the cellular context and multiple interacting pathways.^190^ DNA methylation and histone modifications synergistically can impact gene expression in gastrointestinal cancer. Tumor suppressor genes undergo methylation along with repressive histone marks, forming silent chromosomal structures known as heterochromatin, which suppress gene transcription. Conversely, repetitive sequences and transposons exhibit DNA hypomethylation accompanied by active histone marks, leading to the formation of open chromosomal structures called euchromatin and the abnormal activation of these elements^191^ (Figure 4). Despite this, DNA methylation and histone modification can also exert opposing effects. Funata et al.^192^ discovered that following EBV infection, while many genes underwent methylation, some of these genes were not completely silenced, depending on their classification as DNA methylation‐sensitive or DNA methylation‐resistant genes. DNA methylation‐resistant genes retained gene expression following EBV infection, with TSSs shielded from DNA methylation by active histone marks (H3K4me3 and H3K27e3), including *MLH1*, *MSH2*, and others. Therefore, histone marks may redistribute EBV‐associated DNA methylation.

Exploring the relationship between DNA methylation and histone modification in the early stages and disease progression of gastrointestinal cancer is a crucial pursuit, as it offers insight into the genetic and epigenetic mechanisms of gastrointestinal cancer development and enables the formulation of more efficacious treatments. Such clinical studies can be complemented by established in vitro and in vivo experiments, such as specific epigenetic inhibitor treatments in cell lines, organoids, and animal disease models. Coregulation by multiple modifications occurs in the *MLH1* promoter of both SGC‐7901 and MGC‐803. However, the DNA demethylating agent azacytidine is the sole restorative agent of MLH1 gene expression, as HDACi aspergillin A is unable to replicate its effect. This suggests that DNA methylation plays an indispensable role in gene silencing.^193^ The protein tyrosine phosphatase receptor‐type O (PTPRO) is involved in the tyrosine phosphorylation of histones. Due to high promoter methylation, it is silenced and associated with various malignant tumors. Studies utilizing cell and animal models, along with patient samples, suggest that PTPRO can exert inhibitory effects on ESCC.^194^ The coordinated regulation of DNA methylation and histone modifications is an important integral aspect of the epigenetic landscape, with specific associations between these factors necessitating further functional study.

## 6 CLINICAL APPLICATION OF EPIGENETICS IN GI CANCERS

### 6.1 Diagnosis

Abundant methylation of oncogenes and tumor suppressor genes is present in the early stages of malignant transformation. This suggests that DNA methylation can serve as a biomarker for the diagnosis of malignant tumors (Table 1). Recently, a new method for early diagnosis of gastrointestinal cancers based on DNA methylation has been developed. Researchers conducted an exhaustive analysis of genome‐wide DNA methylation patterns across 1781 tumors and adjacent normal tissues, discerning regions exhibiting distinctive methylation patterns. Employing sophisticated algorithms, they crafted a predictive framework encompassing colorectal, gastric, and ECs, yielding respective accuracies of 98, 94, and 90%.^195^ EC, a type of cancer with distinct characteristics in China, has seen a continuous rise in incidence in recent years, necessitating noninvasive diagnostic tools. Hope is brought by DNA methylation markers detected in plasma. Researchers found that individual DNA methylation markers cannot effectively diagnose EC. As a better approach, they curated a marker panel comprising *FER1L4*, *ZNF671*, *ST8SIA1*, *TBX15*, and *ARHGEF4*, achieving a diagnostic accuracy of 74%. Remarkably, this accuracy remains unaffected by variables such as age, gender, and smoking habits.^196^ *JAM3* is frequently methylated in human EC and can serve as an early detection marker for EC.^197^

**TABLE 1:** Examples of DNA methylation markers of prediction, diagnostic, and prognostic value in gastrointestinal cancer.

Biomarker	Type	Detected in patients with gastric cancer (%)	Detected in control donors (%)	References
DAPK	Prediction	44.8	21.4	^198^
BNIP3	Prediction	49	21	^198^
PYCARD	Prediction	48	22	^199^
RPRM	Diagnostic	95.3	9.7	^200^
RUNX3	Diagnostic	45	8	^201^
MGMT	Diagnostic	70	36	^202^
Reprimo	Diagnostic	95.7	9.7	^200^
SLC19A3	Diagnostic	85	15	^203^
CDKN2A	Diagnostic	26.9	0	^204^
RASSAF1A	Diagnostic	34	0	^205^
P16	Diagnostic	51.9	0	^206^
P15	Diagnostic	55.6	0	^206^
DKK3	Prognosis	67.6	34.6	^207^
TIMP3	Prognosis	63	4.3	^208^
PAX6	Prognosis	0.3−108.1	5.0−54.2	^209^
MINT2	Prognosis	44.6	3.3	^210^
PCDH10	Prognosis	83.2	5.45	^211^
XAF1	Prognosis	83.2	0	^212^
TFP12	Prognosis	80.9	0	^213^
VAV3	Prognosis	54.2	0	^214^

Belated diagnosis contributes significantly to the high mortality rates associated with GC. Presently, upper gastrointestinal endoscopy is regarded as the gold standard for diagnosing GC,^215^ with imaging serving as a supplementary tool for determining the pathological subtype. Through comprehensive methylation analysis, the abnormal methylation patterns of CGIs within promoter regions are emerging as promising molecular biomarkers for GC diagnosis. Various DNA methylation markers with distinct specificity and sensitivity have been discovered in the plasma, serum, gastric juice, and stool samples of GC patients.^216^ In the serum of individuals diagnosed with GC, methylation of various tumor suppressor genes, such as *DAPK*, *CDH1*, *CDKN2A*, and *CDKN2B*, has been observed, with *CDKN2A* methylation aiding in the prediction of malignancy potential in gastric dysplasia.^217^ The hypermethylation of the *RPRM* gene and *MINT25* (an alternative promoter of *CABIN1*) has been detected in gastric lavage fluid and plasma of GC patients.^218^

Colonoscopy and other invasive methods are considered the gold standard for diagnosing CRC. Serum carcinoembryonic antigen is also utilized in CRC diagnosis; however, its sensitivity and specificity are relatively low.^219^ Luo et al.^220^ conducted a meticulous examination of DNA methylation patterns within hematologic specimens procured from diverse and expansive patient cohorts. Through this extensive analysis, they ascertained and subsequently validated a methylation‐centric diagnostic scoring system designed for the identification of CRC patients. Notably, cg10673833 emerged as the epitome of diagnostic efficacy, boasting a sensitivity of 89.7% (95% CI, 0.727–0.978) and a specificity of 86.8% (95% CI, 0.849–0.884).^220^ Due to a plethora of recent studies, the United States Food and Drug Administration (US FDA) has approved a DNA methylation‐based screening analysis for CRC.

### 6.2 Treatment

In summary, targeting epigenetic modifications shows potential for treating gastrointestinal tumors. The reversibility of epigenetic reprogramming renders epigenetic drivers as promising therapeutic targets for intervening in and counteracting aberrant epigenetic modifications. Epigenetic interventions encompass HAT inhibitors (HATis), HDACis, and DNMT inhibitors (DNMTis). Diverse assays for epigenetic targets in gastrointestinal tumors are currently underway (Table 2). gastrointestinal tumors epigenetic reprogramming fosters the reactivation of tumor suppressor genes, suppresses proto‐oncogene expression, and heightens tumor cell responsiveness to chemotherapy, radiotherapy, and immunotherapy. In summary, targeting epigenetic modifications shows potential for treating gastrointestinal tumors.

**TABLE 2:** Epigenetic modification‐related clinical trials.

Drug	Mechanism of action	Sample size	Current status	Phase	NCT
Azacitidine	DNMTi	70	Terminated	2	NCT02959437
Vorinostat	HDACi	45	Completed	1/2	NCT01045538
CUDC‐101	HDACi	47	Completed	1	NCT01171924
Tucidinostat	HDACi	87	Recruiting	2	NCT05163483
CHR‐3996	HDACi	40	Completed	1	NCT00697879
KA2507	HDAC6i	20	Completed	1	NCT03008018
Hydralazine	DNMTi	15	Completed	2	NCT00404508
Vorinostat	HDACi	72	Active, not recruiting	1	NCT01023737
JBI‐802	LSD1i/HDAC6i	126	Recruiting	1/2	NCT05268666
aza‐TdC	DNMTi	50	Recruiting	1	NCT03366116
CUDC‐907	HDACi	43	Completed	1	NCT02307240
5‐Azacytidine	DNMTi	31	Active, not recruiting	1	NCT03206021
Entinostat	DNMTi	21	Completed	1	NCT02780804
NTX‐301	DNMT1	125	Recruiting	1/2	NCT04851834
TdCyd	DNMTi	27	Suspended	1	NCT02423057
Chidamide	HDAC1, 2, 3, 10i	100	Recruiting	1/2	NCT05320640
MG 98	DNMTi	19	Completed	1	NCT00003890
CC‐486	DNMTi	169	Completed	1	NCT01478685

*Data sources*—Home—ClinicalTrials.gov.

#### 6.2.1 DNA methylation inhibitors

DNA methylation leads to transcriptional repression, primarily associated with heterochromatin. DNMTis suppress DNA methylation, resulting in widespread gene hypomethylation in cells. The pioneering DNMTis, 5‐azacytidine and decitabine, were developed in the 1970s as cytosine nucleoside analogs that could be incorporated into newly synthesized DNA but were incapable of accepting a methyl donor at the 5′ position of the pyrimidine ring, thereby depleting cellular DNMT1.^86^ Gemcitabine, a second‐generation DNMTi, also known as 5‐aza‐2′‐deoxycytidine, is a more selective drug that induces gene activation and cell differentiation in vitro by inducing DNA hypomethylation. It has been employed in combination with other anticancer agents to treat GC.^221^ Research findings have indicated that azacytidine possesses the capability to diminish DNA methylation levels and impede the proliferation of GC cells, particularly those displaying the CIMP.^222^, ^223^ In a Mongolian gerbil model, azacytidine was found to prevent Helicobacter pylori‐induced GCs with no overt adverse effects, except for testicular shrinkage.^224^

DNMTis are currently under investigation for their therapeutic potential in CRC. C‐terminal Src kinase, a kinase with limited current research, has been reported to inhibit *SRC* activation in CRC cells by reducing CHK protein levels. 5‐Aza‐CdR inhibits the proliferation, colony growth, and invasion of CRC cells caused by the downregulation of CHK protein expression.^225^ It is noteworthy that DNMTis currently confront formidable clinical challenges, encompassing low drug responsiveness, drug resistance, and side effects. This emphasizes the need for a better understanding of DNMTi molecular targets. Using the CRC HCT116 cell line as a model, researchers identified 638 novel CpGs, with high methylation of CpGs promoting the therapeutic effect of cytidine analogs.^226^ Furthermore, the DNMT1 gene undergoes deletion to variable extents in approximately 9% of human CRCs. This deletion markedly diminishes the cytotoxicity and growth inhibition induced in CRC cells by decitabine, cytarabine, and 5‐azacytidine‐4′‐thio‐2′‐deoxycytidine. Ergo, CRC patients harboring a *DNMT1* gene deletion may exhibit refractoriness to DNMTi interventions.^227^

Similarly, DNMTis represent a novel direction in the treatment of EC. In EC cells, LINC01270 recruits DNMTs DNMT1, DNMT3A, and DNMT3B to activate *GSTP1* promoter methylation, thereby instigating the proliferation, migration, and invasion of EC cells. This activation leads to the proliferation, migration, and invasion of EC cells. The use of the *GSTP1* methylation inhibitor SGI‐1027 can inhibit the progression of EC.^228^ Insulin‐like growth factor‐binding protein 1 (*IGFBPL1*) exerts inhibitory dominion over the proliferation and clonogenicity of EC cells by effectively subduing both in vivo and in vitro PI3K–AKT signaling pathways. *IGFBPL1* undergoes methylation in 47.3% (53 out of 114) of cases afflicted with esophageal dysplasia and 49.1% (246 out of 501) of cases characterized by primary ESCC. Empirical findings corroborate that 5‐AZA‐2′‐deoxycytidine holds the capacity to reinstate or augment the expression of *IGFBPL1* in EC cells, exemplified by KYSE150, KYSE410, and KYSE520.^229^ Importantly, DNMTis can reverse drug resistance in EC cells. Some EC patients treated with TKIs exhibit resistance. Researchers have found that these cancer cells evade apoptosis by consuming large amounts of arginine. The increased dependence on arginine flux indirectly leads to genome‐wide hypermethylation, providing cancer cells with a proliferative advantage. The use of the DNA methylation inhibitor decitabine can thus reverse TKI resistance.^230^

#### 6.2.2 HDAC inhibitors

HDACis, as a novel class of anticancer drugs, have the capacity to regulate EMT, restraining the migration and invasion of cancer cells. Several US FDA‐approved HDACis drugs, such as belinostat, panobinostat, lomostat, and vorinostat, have been developed for treating multiple myeloma and cutaneous T‐cell lymphoma.^231^ Suberoylanilide hydroxamic acid, an HDACi applied in cancer therapy, lacks specificity due to its multitarget nature, resulting in poor cytotoxic effects. Researchers have ingeniously designed and developed TC24, a drug selectively inhibiting HDAC6. TC24 exhibits robust antiproliferative and antimigratory capabilities against GC cells, while causing no significant cytotoxic effects on normal gastric GES‐1 cells.^232^ Furthermore, an auspicious array of 1,3‐diphenyl‐1,2,4‐triazole‐terminated HDAC6 inhibitors has been conceived, synthesized, and substantiated in a scholarly investigation. In this, compound 9r emerges as the epitome of specificity, showcasing a selectivity 128 times greater for HDAC6 in comparison with HDAC1. Compound 9r not only induces apoptosis but also impedes metastasis in GC cells without conspicuous side effects.^233^ HDAC3 serves as a positive regulator of GC cell proliferation and migration. Hesperidin significantly reduces HDAC3 activity at the catalytic tyrosine 298 residue and, simultaneously, markedly inhibits HDAC3 expression by suppressing NFκBp65/CEBPβ signaling, thereby impeding the metastatic spread of GC cells.^234^

The cancer database indicates that HDAC6 also plays a role in EC, significantly correlating with patients' survival time.^235^ ACEY‐1215, alternatively known as Ricolinostat, emerges as a discerning inhibitor targeting HDAC6, manifesting an inhibitory potency exceeding 10‐fold compared with HDAC1, HDAC2, and HDAC3. This compound elicits apoptosis in EC cells by impeding the G2/M phase through the inhibition of the ERK pathway and PI3K/AKT/mTOR signaling.^236^ HDAC1 and HDAC2 are overexpressed in ESCC tissues and are associated with the clinical pathological features of ESCC patients. Scientific evidence denotes that the HDACi MS‐275 profoundly diminishes the expression levels of both HDAC1 and HDAC2. Through the blockade of the PI3K/Akt/mTOR pathway, it effectively curtails the proliferation and clonogenicity of ESCC cells, both in vitro and in vivo. The efficacy of this inhibitor demonstrates a reliance on concentration, signifying its promising potential as a therapeutic modality for ESCC.^237^

*NEDD9* undergoes dynamic alterations in activity in CRC. Broad‐spectrum HDACis enhance the acetylation of H3K9 on the *NEDD9* promoter by inhibiting HDAC4 activity. Consequently, this augmentation leads to an increased expression of *NEDD9*, facilitating FAK phosphorylation and restraining the metastatic potential of CRC.^238^ On one facet, BEBT‐908 fosters the manifestation of iron death signaling by mitigating the hyperacetylation of *P53*, thereby orchestrating the demise of cancer cells. On the other facet, it incites a proinflammatory milieu within the tumor microenvironment, eliciting the host's antitumor immune response and suppressing the proliferation and metastasis of CRC.^239^

#### 6.2.3 Histone methylation inhibitors

HATis can effectively target histone modifications and are used for the treatment of GC. DZNep effectively depletes *EZH2* and inhibits the H3K27me3 marker in GC cells.^240^ As a consequence, the ubiquitination of *p53* is hindered, leading to *p53* stabilization and subsequent activation of the downstream *p53* pathway, ultimately culminating in apoptosis and cell cycle arrest. Moreover, Emran et al.^241^ have reported that a decrease in the active histone marker H3K4me3 and an increase in the inhibitory histone marker H3K9me3 can lead to multidrug tolerance. However, the resistance was found to be reversible upon the use of H3K9me3 inhibitors SETDB1/2.

Abundant research indicates that various PTMs of the Forkhead family transcription factor *FOXO1* significantly regulate its activity. In CRC, researchers have discovered that G9a can methylate the K273 residue of *FOXO1* both in vivo and in vitro. This diminishes the stability of the *FOXO1* protein, promoting cancer cell proliferation, and inhibiting apoptosis. Additionally, the G9a‐specific inhibitor BIX‐01294 can suppress the methylation of *FOXO1*, thereby modulating cancer cell proliferation and apoptosis, exerting anticancer effects.^242^ The lysine methyltransferase SUV420H2 promotes H4K20me3, thereby maintaining heterochromatin compaction and preventing the formation of R‐loops. Within the context of CRC, inhibitors of HDT showcase the ability to curtail chromatin accessibility, thereby eliciting a reduction in the growth of subcutaneous tumors in murine models.^159^ LSD1/KDM1A has the ability to regulate the methylation of H3K4/9 and serves as a promising epigenetic target for anticancer purposes. Currently, multiple LSD1 inhibitors have been developed, although only two reversible LSD1 inhibitors, CC‐90011 and SP‐2577, are in the clinical stages for treating EC.^243^

#### 6.2.4 Combination therapy

The intricate epigenetic landscape of gastrointestinal cancer encompasses a diverse array of processes, thereby hinting at the potential of combination therapies that target various key epigenetic drivers, offering promising prospects for precise patient treatment. In this regard, a dual‐action small molecule drug, 4SC‐202, which targets HDAC1/2/3 and LSD1, is currently being investigated in clinical trials. Nonetheless, the challenge with epigenetic therapy lies in the highly heterogeneous and intricate microenvironment of gastrointestinal cancer. Merely targeting DNMTis, HDACis, and HATis may not be sufficient for activating the expression of tumor suppressor genes. An emerging body of literature has demonstrated that the best approach to utilizing epigenetic drugs is to combine them with chemoradiotherapy or immunotherapy. The primary reasons for the persistent challenges in treating ESCC include chemotherapy resistance and an unfavorable prognosis associated with EMT. In vivo and in vitro experiments validate that the depletion of mitochondrial DNA and depolarization of the mitochondrial membrane potential induce EMT. DNMTis can suppress EMT and enhance the chemosensitivity of ESCC cells with depleted mtDNA. This suggests that the combination of DNMTis and chemotherapy holds promise as a novel approach to cure ESCC.^244^

According to a report, the proliferative activity of gastric carcinoma cell lines (OCUM‐2 M, OCUM‐12, and MKN‐45GC) treated with irradiation and decitabine was significantly lower than that of cells treated with irradiation alone (*p *< 0.05). This phenomenon could potentially be attributed to decitabine's proficiency in augmenting the expression of gene clusters including *P53*, *RASSF1*, and *DAPK*, thereby orchestrating cell cycle arrest and apoptosis.^245^ Furthermore, evidence suggests that combining decitabine with conventional chemotherapy can make tumor cells more sensitive to chemotherapy. As an illustrative instance, the coadministration of decitabine and 5‐FU holds the capacity to mitigate the advancement of gastric carcinoma by demethylating and amplifying the susceptibility of gastric carcinoma cells towards 5‐FU.^246^ In CRC, *CDKN2A* promoter methylation activates the interferon pathway, increasing the expression of *PDL1*. Researchers have discovered that combined treatment with 5‐aza‐2′‐deoxycytidine and anti‐PD‐L1 more effectively improves the survival rate of tumor‐bearing mice than blocking either pathway alone.^247^ *RTP4* is a interferon‐stimulated genes. Studies have revealed that in anti‐PD‐1‐resistant subclones of CRC, RTP4 is silenced by the H3K9 methylation, affecting the recruitment of cancer cells to T lymphocytes. This suggests that the combined application of histone methylation inhibitors and anti‐PD‐1 may have a synergistic effect in inhibiting CRC.^248^

### 6.3 Prognosis

Aberrant DNA methylation analysis stands as a method for detecting prognostic biomarkers, owing to its stability and autonomy (Table 1).^249^ Copious investigations accentuate the genes and loci displaying anomalous methylation as prognostic markers for BE and EAC.^18^ Scholars, utilizing the Survival R package, evaluated the prognostic value of methylation‐driven genes. Notably, *ABCD1*, *SLC5A10*, *SPIN3*, *ZNF69*, and *ZNF608* emerged as independent prognostic indicators for ESCC (*p *< 0.05).^250^ A thorough examination, encompassing 1633 samples of EC, uncovered a substantial correlation between heightened methylation levels of *CDH1* and escalated EC risk (OR = 10.40, 95% CI = 6.29−17.18). The overall sensitivity and specificity of *CDH1* methylation in EC prognosis were discerned as 0.57 (95% CI = 0.39–0.74) and 0.89 (95% CI = 0.81−0.94), respectively.^80^

Research has indicated that the overall survival rate of GC patients can be predicted through CIMP‐H (*p *= 0.001), showing an association with improved survival rates. However, it is not an independent prognostic factor for the postoperative prognosis of GC. And methylation of the *MGMT* gene has been linked to a poor prognosis in GC patients.^51^ Studies have demonstrated that hypermethylation of *PCDH10* is significantly associated with a worse survival rate in patients with GC.^251^ Furthermore, several studies have demonstrated that promoter hypermethylation of *TMBI3*, *MINT2*, *DKK‐3*, *VAV3*, *TFP12*, *XAF1*, *PAX6*, and other genes are significantly associated with poor clinical outcomes in GC patients. Notably, recent research has revealed that hypermethylation of *BNIP3* and *DAPK* genes serve as a predictive marker.^198^

Among all epigenetic biomarkers, the CIMP status in CRC stands out as the most promising prognostic indicator. In a cohort of 206 stage III CRC patients, researchers found a correlation between CIMP‐positive status and lower survival rates.^252^ Nevertheless, beyond CIMP, the MSI status assumes a pivotal role as a substantial confounding variable. Investigations have illuminated the impact of the tumor's MSI status on the prognosis of CRC patients with CIMP positivity.^253^ DNA methylation leads to a significant upregulation of *CysLT1* and downregulation of *CysLT2* in primary CRC tumor tissues. Interestingly, *CysLT1* significantly predicts adverse outcomes in terms of overall survival (HR = 2.14, *p *= 0.03), while *CysLT2* significantly predicts adverse outcomes in terms of disease‐free survival (HR = 2.88, *p *= 0.03).^254^ *CXCL14*, situated among the most evolutionarily conserved constituents of the chemokine family, manifests pronounced transcriptional repression in clinical colon cancer specimens attributable to promoter methylation. Empirical studies have delineated a correlation between the methylation levels of *CXCL14* and tumor prognosis, positioning it as a prospective biomarker for prognosticating the trajectory of CRC.^255^

Epigenetic modifications are crucial in the diagnosis, precise treatment, and prognosis of gastrointestinal cancer. However, some concerns must not be overlooked. First, epigenetic reprogramming is reversible, which means that re‐methylation and activation may occur after drug treatment is interrupted.^256^ Moreover, the specificity of epigenetic therapy has drawn attention, and certain studies have reported nonspecific gene effects in normal cells. Furthermore, the discernment surrounding the selectiveness of epigenetic therapy has sparked scholarly contemplation; certain investigations have illuminated the existence of nonspecific genetic impacts in the normal cellular. Several studies have demonstrated that the utilization of 5‐Aza and decitabine increases gene mutation rates in patients and the risk of chromosomal rearrangements.^257^ Therefore, extensive clinical research is required to investigate the effects of epigenetic therapy and resolve these issues.

## 7 CONCLUSION

In summary, epigenetic reprogramming, encompassing specific DNA methylation and histone modifications, exerts a profound influence on the expression of numerous downstream cancer‐related genes, thereby assuming a pivotal role in the trajectory of gastrointestinal cancer development. Notably, gastrointestinal cancer is often characterized by a dualistic epigenetic profile: widespread hypomethylation across the genome juxtaposed with hypermethylation at CGIs. Furthermore, aberrant histone modifications contribute to gene dysfunction and inappropriate gene activation within the gastrointestinal cancer context. Importantly, it is worth highlighting the interdependent progression of DNA methylation and histone modifications. A substantial body of evidence attests to their synergistic orchestration in sculpting silent or accessible chromatin states among individuals afflicted with gastrointestinal cancer.

In parallel, epigenetic biomarkers have emerged as significant indicators for the diagnosis, personalized therapy, and prognostication of gastrointestinal cancer. DNA methylation markers present in patients’ plasma, serum, gastric juice, and stool samples have gained extensive utilization in the realm of cancer diagnosis and prognosis. HATis, HDACis, and DNMTis have also been developed, targeting aberrant epigenetic pathways and demonstrating efficacy in gastrointestinal cancer treatment. The amalgamation of epigenetic agents with chemotherapy and/or immunotherapy compounds has undeniably emerged as the prospective treatment paradigm for individuals afflicted with gastrointestinal cancer.

While epigenetic reprogramming has emerged as a leading field in gastrointestinal cancer research, the scope of widely investigated genes, when juxtaposed with the vast expanse of the human genome, remains limited. Moving forward, the utilization of high‐throughput sequencing technologies holds the potential to unveil novel epigenetic phenomena. This pursuit bears profound significance, encompassing the diagnosis of gastrointestinal cancer onset, prognosis prediction, and the formulation of more precisely targeted therapeutic strategies. Furthermore, scientists are poised to not only delineate the epigenetic landscape of gastrointestinal cancer but also unravel the intricate interplay between genetics and epigenetics. Recent advancements in research have underscored the universal occurrence of epigenetic information loss across biological entities, thereby instigating cellular aging.^258^ The exploration of dynamic epigenetic alterations within tumor cells holds the promise of emerging as a novel therapeutic avenue for individuals afflicted with gastrointestinal cancer.

## AUTHOR CONTRIBUTIONS

Yingjie Wang, Hongyu Liu, Mengsha Zhang, and Jing Xu wrote the main manuscript text and prepared Figures 1, 2, 3, 4 and Tables 1 and 2; Chong Chen proved the conceptuailzation. All authors have read and approved the final manuscript.

## CONFLICT OF INTEREST STATEMENT

The authors declare no conflict of interests.

## ETHICS STATEMENT

Not applicable.

## Data Availability

Not applicable.
